# Antitumor Effects of Delta (9)-Tetrahydrocannabinol and Cannabinol on Cholangiocarcinoma Cells and Xenograft Mouse Models

**DOI:** 10.1155/2022/6477132

**Published:** 2022-11-21

**Authors:** Surang Leelawat, Kawin Leelawat, Thunyatorn Yimsoo, Thaniya Wunnakup, Chaowalit Monton, Nanthaphong Khamthong, Fameera Madaka, Athip Maha, Thanapat Songsak

**Affiliations:** ^1^Medicinal Cannabis Research Institute, College of Pharmacy, Rangsit University, Pathum Thani 12000, Thailand; ^2^Department of Pharmacognosy, College of Pharmacy, Rangsit University, Pathum Thani 12000, Thailand; ^3^Department of Surgery, Bumrungrad International Hospital, Bangkok 10110, Thailand; ^4^Laboratory Animal Center, Thammasat University, Pathum Thani 12120, Thailand; ^5^Drug and Herbal Product Research and Development Center, College of Pharmacy, Rangsit University, Pathum Thani 12000, Thailand; ^6^College of Oriental Medicine, Rangsit University, Pathum Thani 12000, Thailand

## Abstract

Cholangiocarcinoma (CCA) is a very aggressive tumor. The development of a new therapeutic drug for CCA is required. This study aims to evaluate the antitumor effect of ∆^9^-tetrahydrocannabinol (THC), the major psychoactive component of marijuana (*Cannabis sativa*), and cannabinol (CBN), a minor, low-psychoactive cannabinoid, on CCA cells and xenograft mice. THC and CBN were isolated, and their identities were confirmed by comparing ^1^H- and ^13^C-NMR spectra and mass spectra with a database. Cell proliferation, cell migration, and cell apoptosis assays were performed in HuCCT1 human CCA cells treated with THC or CBN. The phosphorylation of signaling molecules in HuCCT1 cells was detected. To determine the effects of THC and CBN in an animal model, HuCCT1 cells were inoculated subcutaneously into nude mice. After the tumors reached an appropriate size, the mice were treated with THC or CBN for 21 days. Tumor volumes were monitored and calculated. The ^1^H- and ^13^C-NMR data of THC and CBN were almost identical to those reported in the literature. THC and CBN significantly inhibited cell proliferation and migration and induced apoptosis in HuCCT1 cells. The phosphorylation of AKT, GSK-3*α*/*β*, and ERK1/2 decreased in HuCCT1 cells treated with THC or CBN. CCA xenograft mice treated with THC showed significantly slower tumor progression and smaller tumor volumes than control mice. THC and CBN induced apoptosis in CCA by inhibiting the AKT and MAPK pathways. These findings provide a strong rationale for THC and CBN as therapeutic options for CCA.

## 1. Introduction

Cholangiocarcinoma (CCA) is an adenocarcinoma of the epithelial cells lining the intrahepatic and extrahepatic bile ducts [1]. It is a rare tumor worldwide but one of the most common cancers in northeast Thailand [2]. The tumor is generally found to invade local organs and regional lymph nodes. CCA is highly resistant to chemotherapy, surgical resection is the curative treatment option. The five-year survival rate following surgical resection of CCA is only 30% [3]. Platinum-based chemotherapy and gemcitabine is the standard systemic treatment for CCA; however, it provides only a slight survival advantage. Therefore, identifying an effective treatment for CCA patients is crucial.

Cholangiocarcinogenesis is composed of a complex relationship of extracellular ligands, upregulated cell surface receptors, and dysregulated intracellular protein phosphorylation signaling pathways, leading to cell proliferation, survival, and metastasis [4]. Chronic inflammation and/or cholestasis comprise the majority of cholangiocarcinoma risk factors. Inflammatory mediators such as IL-6 and TNF activate many signaling pathways, including JAK-STAT, p38 MAPK, and AKT [5]. Furthermore, the abnormal regulation of kinase and phosphatase signaling pathways plays a principal role in oncogenic pathway activation in numerous cancers [6, 7]. Previous research demonstrated that ERK signaling modulation inhibits CCA proliferation and metastasis [8]. Moreover, the suppression of VEGFR2 phosphorylation levels reduces the PI3K/AKT signaling pathway, resulting in alterations in CCA cell proliferation, apoptosis, and invasion [9]. The manipulation of protein phosphorylation or signal transduction may be valuable for CCA therapy.

∆^9^-Tetrahydrocannabinol (THC) is the principal component of marijuana (*Cannabis sativa*). Currently, THC is used medically to treat chemotherapy-induced nausea and vomiting in cancer patients and anorexia in AIDS (acquired immune deficiency syndrome) patients [10, 11]. Considerable research has been conducted on the potential use of cannabis in cancer patients as an anticancer and symptomatic relief therapy [12]. THC stimulates cannabinoid receptors (CB1 and CB2 receptors) and induces an endoplasmic reticulum (ER) stress-related response that inhibits the AKT–mammalian target of the rapamycin complex 1 (mTORC1) axis and induces autophagy. THC inhibits AKT, which induces cycle arrest and apoptosis in breast cancer cells and melanoma [13]. A previous study demonstrated that CCA cell lines and surgical specimens from CCA patients expressed cannabinoid receptors. THC inhibits CCA cell proliferation and induces CCA cell apoptosis via the inhibition of AKT and MEK1/2 phosphorylation [14]. THC appears to be a potential therapeutic agent for treating CCA. However, THC causes symptoms of mental illness and cognitive impairment [15]. THC can potentially lead to dependence and behavioural disturbances and may enhance the risk of psychotic conditions [16, 17]. Therefore, the discovery of compounds comparable to THC with fewer psychotropic side effects is essential for CCA therapy.

Cannabinol (CBN) is a nonenzymatic degradative byproduct of THC. CBN is derived after the prolonged storage of cannabis, especially at higher temperatures. Compared to THC, CBN shows low binding potency to the CB1 receptor and has mild psychoactive effects [18, 19]. However, the anticancer activity and molecular mechanism of CBN have yet to be identified, particularly in vivo.

This research studies the effects of THC and CBN on human CCA cell apoptosis and tumor volume in xenograft mice. The molecular mechanism involved in the signal transduction of human CCA cells treated with THC and CBN, including protein phosphorylation, was investigated.

## 2. Materials and Methods

### 2.1. Chemicals and Materials

RPMI 1640 medium and fetal bovine serum were purchased from Gibco (Grand Island, NY, USA). Polyclonal antibodies against protein kinase B (AKT) (phosphorylated at Ser473), glycogen synthase kinase 3 alpha/beta (GSK-3*α*/*β*) (phosphorylated at Ser21/9), p44/42 mitogen-activated protein kinase (MAPK) (ERK1/2) (phosphorylated at Thr202/Tyr204), caspase 3, cleaved caspase 3, poly (ADP-ribose) polymerase (PARP [46D11]), cleaved PARP (Asp214), caspase 3, cleaved caspase 3 (Asp175), and *β*-actin were obtained from Cell Signaling Technology (Beverly, MA, USA). LY 294002, U0126, radioimmunoprecipitation assay (RIPA) buffer and protease inhibitor cocktail were purchased from Cell Signaling Technology (Beverly, MA, USA). Culture plates and flasks were obtained from Falcon (Corning, NY, USA). Matrigel basement membrane matrix was purchased from Corning (NY, USA). Tween 80 was purchased from P. C. Drug Center (Bangkok, Thailand).

### 2.2. Cell Cultures

The human CCA cell line HuCCT1 (Riken Bioresource Research Center, Japan) was grown in RPMI 1640 medium supplemented with 10% fetal bovine serum at 37°C in a 5% CO_2_ humidified atmosphere. For signal transduction experiments, cells were starved overnight in a serum-free medium.

### 2.3. Isolation and Identification of THC and CBN

THC and CBN were isolated in-house at the College of Pharmacy from dried cannabis samples by column chromatography and preparative HPLC. The identity of the compounds was confirmed by comparing their ^1^H- and ^13^C-NMR data with those in the literature and matching their mass spectral data to those in the NIST database. The purity was quantitatively analyzed by HPLC (Prominence UFLC, Shimazu, Japan) (mobile phase: (ammonium formate: acetonitrile), gradient elution, flow rate 1.0 mL/min, column: XBridge C18, detection at 228 nm).

### 2.4. Cell Proliferation Assay

HuCCT1 cells were seeded in 96-well plates at a density of 10,000 cells/well. After 24 h of incubation, cells were treated with either THC or CBN at concentrations of 10 *μ*M–100 µM, LY294002 [a selective inhibitor of phosphatidylinositol 3 (PI3) kinase], U0126 (a selective inhibitor of ERK1/2) at concentrations of 10 *μ*M and 20 *μ*M, or vehicle. After the treated cells were incubated for 24 h or 48 h, 50 mL of MTT [(3, 4, 5-dimethyl thiazol-2-yl)-2-5-diphenyltetrazolium bromide] (Sigma-Aldrich, Germany) was applied in each well at a concentration of 0.5 mg/mL. The reaction was detected at 590 nm using a Benchmark Plus microplate reader (Bio-Rad, CA, USA).

### 2.5. Cell Migration

HuCCT1 cells were seeded in 24-well plates (3 × 10^5^ cells/well) and cultured for 24 h to form a confluent monolayer. A sterile 10 *μ*L micropipette tip was used to create a straight-edged cell-free zone across the cell monolayer in each well. Then, the monolayer was washed with 500 *μ*L PBS to remove the detached cells. Cells were treated with 15 *μ*M of THC or CBN for 6 h–12 h. The scratch closure was imaged under phase contrast microscopy (Nikon Eclipse TS100, USA) at 4x magnification. The distance of wound width was analyzed using ImageJ digital imaging processing software (ImageJ 1.48v, National Institutes of Health, Bethesda, MD, USA). The percentage of cell migration was calculated as the percentage of wound closure: % wound closure = (A0h–A∆h)  × 100/A0h, where A0h is the area of the wound measured immediately after scratching and A∆h is the area of the wound measured *h* hours after the scratch is performed. Data of three replicates are presented as mean ± SD.

### 2.6. Detection of Cell Apoptosis

Cell apoptosis was determined by flow cytometric analysis. HuCCT1 cells were seeded in culture flasks. After 18 h of incubation, cells were treated with THC or CBN at concentrations of 10 *μ*M–20 *μ*M or with a vehicle. Treated cells were collected after 18 h. An annexin V : FITC assay kit (Bio-Rad, CA, USA) was used following the manufacturer's protocol. Adherent cells were collected, resuspended in 200 *μ*L of binding buffer containing 5 *μ*L annexin V : FITC and incubated for 10 min in the dark at room temperature. Cells were then washed, resuspended in 200 *μ*L of binding buffer containing 10 *μ*L of propidium iodide solution, and analyzed by flow cytometry (BD FACSVerse Flow Cytometer, BD Biosciences, USA).

The numbers of early and late apoptotic cells are shown in the lower right quadrant and upper right quadrant of the histograms, respectively. The lower left quadrants indicate living cells.

### 2.7. Detection of Protein Phosphorylation in Signaling Pathways

To detect signaling pathways, 1 × 10^7^ HuCCT1 cells were seeded in culture flasks overnight. Cells were then treated with THC or CBN (at concentrations of 20 *μ*M) or a vehicle. After 18 h of incubation, the cells were analyzed by a Proteome Profiler Array Human Phosphokinase Array Kit (R&D Systems, Inc., USA) following the manufacturer's instructions. Briefly, cell lysate was incubated overnight with the human phosphokinase array membrane. The membrane was washed to remove unbound proteins and then incubated with a cocktail of biotinylated detection antibodies. Streptavidin-HRP and chemiluminescent detection reagents were applied. The chemiluminescent signal was determined by UVP ChemStudio (Analytik, Jena, Germany) and analyzed by ImageJ digital imaging processing software.

### 2.8. Western Blot Analysis

HuCCT1 cells were seeded in a six-well culture plate at a density of 5 × 10^5^ cells/well, then treated with THC or CBN (at concentrations of 10 *μ*M and 20 *μ*M) or a vehicle for 18 h. Treated cells were collected, washed with PBS, and lysed in RIPA buffer containing a 1% protease inhibitor cocktail. Western blot analyses were performed as previously reported [14]. The blots were probed with antibodies against phosphorylated AKT, GSK-3*α*/*β*, ERK1/2, PARP (46D11), cleaved PARP, and *β*-actin. Colorimetric detection of antigen-antibody complexes revealed the target proteins on the membrane using an Opti-4CN Detection Kit (Bio-Rad, CA, USA) following the manufacturer's instructions. Semiquantification of the protein bands was analyzed using the ImageJ digital imaging processing software. The expression of each analyzed protein was normalized with *β*-actin. The samples were analyzed three times in independent blots.

### 2.9. Animals and Experimental Design

**Figure 1:**
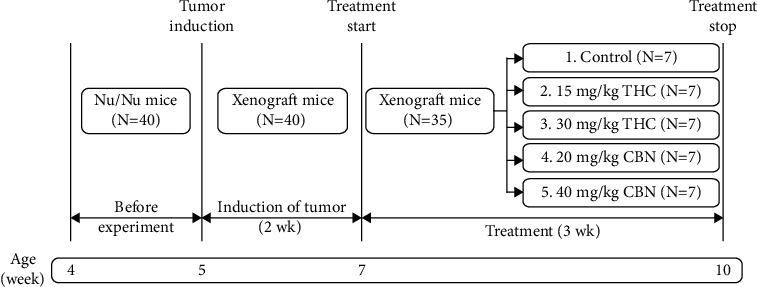
Schematic diagram of experimental design.

Athymic nude (BALB/cAJcl-Nu/Nu) mice were obtained from Nomura Siam International Co., Ltd. (Bangkok, Thailand). The animal protocol was designed to minimize pain and discomfort for the animals. Ethical approval for the study was obtained from Research Institute, Rangsit University (approval number RSEC 05/2559). Four-week-old female mice (weighing 12.7 g–17.0 g) were housed and acclimatized to laboratory conditions (50 ± 20% humidity, 12 h light/12 h dark, 21 ± 1°C) for one week prior to experimentation. All animals had free access to sterile food and water and were cared for under specific pathogen-free conditions. A mixture of 3 × 10^6^ HuCCT1 cells in 100 *μ*L serum-free RPMI 1640 medium and 100 *μ*L matrigel was inoculated subcutaneously into the right flank of each mouse. After tumors reached an average volume of 150 mm^3^, mice were then randomized into five treatment groups of seven animals each: 1% Tween 80 (control); 15 mg/kg THC; 30 mg/kg THC; 20 mg/kg CBN; and 40 mg/kg CBN. THC and CBN injections were prepared in a water base to provide good syringeability and injectability and avoid tissue damage from the hyperosmolarity of the formulation. For the control, 1% Tween 80 was used. The subcutaneous injection treatment was repeated every morning for 21 d (Figure 1). Tumor volumes were monitored twice a week using caliper measurements and were calculated by the following formula: (*L* × *W* × *W*)/2, where *L* is the long diameter of the tumor and *W* is the short diameter of the tumor. The body weights of the mice were recorded twice a week. Tumors were maintained in 10% buffered formalin for histopathological and immunohistochemical examination.

### 2.10. Immunohistochemistry

The specimen sections were deparaffinized and rehydrated. Then, endogenous peroxidase was blocked using a hydrogen peroxide block (Abcam, UK). The specimens were washed, and antigen retrieval was performed by heating in a 10 mM citrate buffer. After protein block solution (Abcam, UK) was applied to reduce nonspecific background staining, the specimens were incubated with primary antibodies overnight at room temperature in a humidified chamber. The antigen-antibody complex was then detected using a Mouse and Rabbit Specific HRP/DAB (ABC) Detection IHC Kit (Abcam, UK), following the manufacturer's instructions. The sections were counterstained with hematoxylin solution (Mayer's modified, Abcam, UK) and dehydrated before mounting with mounting medium (Abcam, UK). As a negative control, the primary antibody was replaced with SignalStain antibody diluent (Cell Signaling Technology, USA). The stained specimens were examined under a Motic BA210 microscope.

### 2.11. Statistical Analysis

The data are described as means, with standard deviations or percentages when appropriate. Data from three or more groups were compared using one-way ANOVA or nonparametric Kruskal–Wallis tests. The Mann–Whitney *U* test was used for comparing two groups, and *P* < 0.05 was considered statistically significant. The rate of xenograft tumor progression (assessed by the number of mice with tumor volumes <400 mm^3^) was evaluated using survival curves and compared using the log-rank test. The sample size was determined using the *G*^*∗*^ power 3.1.9.7 program to detect a difference of 100 mm^3^ of tumor volume between the THC, CBN, and control groups of mice. Using an ANOVA test at a significance level of 0.05 and a power of 0.80, a sample size of seven from each group of mice was selected for the study. The efficacy of tumor induction was estimated to be 90%. Therefore, 40 mice were used in the experiment.

## 3. Results and Discussion

### 3.1. Confirmation of the Identity and Purity of THC and CBN

**Figure 2:**
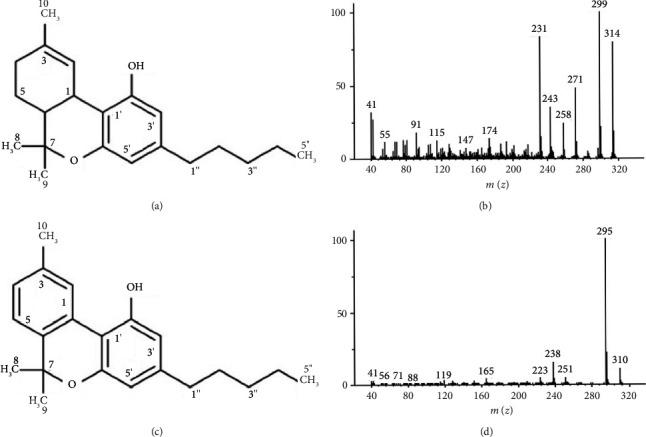
Identification and analysis of THC and CBN. (a) Chemical structure of THC, (b) GC-MS spectrum of THC, (c) chemical structure of CBN, and (d) GC-MS spectrum of CBN.

THC (Figure 2(a)) was isolated as a pale-yellow gum with a molecular ion of *m*/*z* 314 obtained from the GC-MS spectrum (Figure 2(b)). Its ^1^H- and ^13^C-NMR data aligned with the literature [20]. The data are as follows: ^1^H NMR (CDCl_3_, 500 MHz) *δ* 6.31 (1H, *brs*, H-2), 6.27 (1H, *d*, *J* = 1.5 Hz, H-5*′*), 6.14 (1H, *d*, *J* = 1.5 Hz, H-3*′*), 4.92 (1H, *brs*, 2*′*-OH), 3.20 (1H, *d*, *J* = 11.0 Hz, H-1), 2.43 (2H, *td*, *J* = 7.5, 2.0 Hz, H_2_-1*″*), 2.17 (2H, *m*, H_2_-4), 1.91 (1H, *m*, H_a_-5), 1.71 (1H, *m*, H-6), 1.68 (3H, *s*, H_3_-10), 1.55 (2H, *m*, H_2_-2*″*), 1.41 (3H, *s*, H_3_-8), 1.40 (1H, *m*, H_b_-5), 1.30 (4H, *m*, H_2_-3*″*, H_2_-4*″*), 1.09 (3H, *s*, H_3_-9), and 0.88 (3H, *t*, *J* = 7.0 Hz, H_3_-5*″*); ^13^C-NMR (CDCl_3_, 125 MHz) *δ* 154.7 (C, C-2*′*), 154.2 (C, C-6*′*), 142.8 (C, C-4*′*), 134.3 (C, C-3), 123.8 (CH, C-2), 110.0 (C, C-1*′*), 109.0 (CH, C-5*′*), 107.6 (CH, C-3*′*), 77.2 (C, C-7), 45.8 (CH, C-6), 35.5 (CH_2_, C-1*″*), 33.6 (CH, C-1), 31.5 (CH_2_, C-3*″*), 31.2 (CH_2_, C-4), 30.6 (CH_2_, C-2*″*), 27.5 (CH_3_, C-8), 25.0 (CH_2_, C-5), 23.3 (CH_3_, C-10), 22.5 (CH_2_, C-4*″*), 19.2 (CH_3_, C-9), and 14.0 (CH_3_, C-5*″*).

CBN (Figure 2(c)) was isolated as a pale brown gum exhibiting a molecular ion peak at *m*/*z* 310 in the GC-MS spectrum (Figure 2(d)). Its ^1^H- and ^13^C-NMR data were almost identical to those reported in the literature [20]. The data are as follows: ^1^H NMR (CDCl_3_, 500 MHz) *δ* 8.17 (1H, *s*, H-2), 7.15 (1H, *d*, *J* = 8.0 Hz, H-5), 7.07 (1H, *d*, *J* = 8.0 Hz, H-4), 6.44 (1H, *d*, *J* = 1.5 Hz, H-5*′*), 6.29 (1H, *d*, *J* = 1.5 Hz, H-3*′*), 5.25 (1H, *s*, 2*′*-OH), 2.50 (2H, *t*, *J* = 7.5 Hz, H_2_-1*″*), 2.39 (3H, *s*, H_3_-10), 1.62 (2H, *m*, H_2_-2*″*), 1.61 (6H, *s*, H_3_-8, H_3_-9), 1.33 (4H, *m*, H_2_-3*″*, H_2_-4*″*), and 0.90 (3H, *t*, *J* = 7.0 Hz, H_3_-5*″'*); ^13^C-NMR (CDCl_3_, 125 MHz) *δ* 154.6 (C, C-2*′*), 153.0 (C, C-6*′*), 144.5 (C, C-4*′*),136.9 (C, C-3, C-6), 127.6 (CH, C-4), 126.4 (CH, C-2), 122.6 (CH, C-5), 110.8 (C, C-1*′*; CH, C-5*′*), 109.9 (CH, C-3*′*), 108.7 (C, C-1), 77.3 (C, C-7), 35.6 (CH_2_, C-1*″'*), 31.5 (CH_2_, C-3*″'*), 30.4 (CH_2_, C-2*″'*), 27.1 (CH_3_, C-8, C-9), 22.5 (CH_2_, C-4*″'*), 21.5 (CH_3_, C-10), and 14.0 (CH_3_, C-5*″*).

Based on the HPLC chromatograms detected at 228 nm, the compound purities for THC and CBN were found to be 99.31% and 99.76%, respectively.

### 3.2. Effect of THC and CBN on Cell Proliferation and Cell Migration

**Figure 3:**
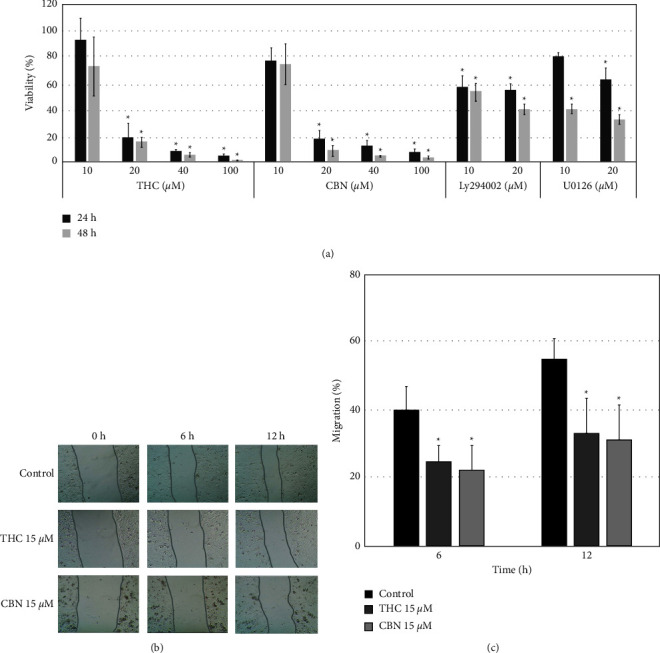
Effect of THC and CBN on cell proliferation and cell migration. (a) THC and CBN significantly inhibited the proliferation of CCA (HuCCT1) cells at 24 h–48 h in a dose-dependent manner. Moreover, HuCCT1 cells were significantly inhibited by LY294002 and U0126 (^*∗*^*P* < 0.05). (b) Inhibition of cancer cell migration was evaluated via wound healing assays of HuCCT1 treated with THC and CBN at a concentration of 15 *μ*M. These images show scratched and recovering wounded areas (marked by grey lines) on confluence monolayers of HuCCT1 cells at 0 h, 6 h, and 12 h, (c) The wound healing assay demonstrated that THC and CBN showed significantly inhibited cell migration compared to the control (^*∗*^*P* < 0.05 versus the control cells).

Cell proliferation assays were performed in the HuCCT1 cells treated with THC or CBN at concentrations from 10 *μ*M to 100 *μ*M or a vehicle (DMSO). THC and CBN concentrations of 10 *μ*M had no significant effect on the inhibition of CCA cell proliferation compared with vehicle-treated cells. However, at high concentrations of THC and CBN (20 *μ*M–100 *μ*M), CCA cell proliferation was significantly inhibited in a dose-dependent manner (Figure 3(a)). The IC_50_ values of HuCCT1 cells for THC and CBN at 24 h of culture were 17.41 ± 1.32 (95% CI, 11.72–23.10) *μ*M and 13.52 ± 1.04 (95% CI, 9.02–18.01) *μ*M, respectively. At 48 h of culture, they were 11.68 ± 2.86 (95% CI, –0.63–23.98) *μ*M and 10.98 ± 2.02 (95% CI, 2.28–19.69) *μ*M, respectively.

The wound healing assay was used to study cell migration. Concentrations of 15 *μ*M of THC and CBN were used because higher doses resulted in massive cell death. The percentage of HuCCA1 cell migration in the control group was 40.18% ± 6.74% at 6 h and 55.21% ± 5.81% at 12 h. Migrations of HuCCT1 cells treated with THC and CBN were significantly decreased at 6 h (24.91 ± 4.69%, *P*=0.026 for THC and 22.18 ± 7.12%, *P*=0.013 for CBN) and 12 h (33.38 ± 10.46%, *P*=0.042 for THC and 31.61 ± 7.12%, *P*=0.031 for CBN) (Figures 3(b) and 3(c)).

### 3.3. Effect of THC and CBN on CCA Cell Apoptosis

**Figure 4:**
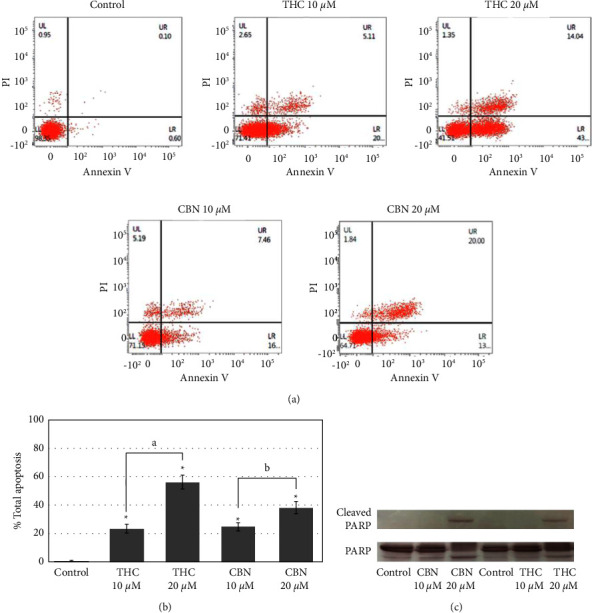
THC and CBN induced apoptosis in CCA (HuCCT1) cells. (a) Flow cytometry results show the percentage of live, apoptotic, and necrotic cells in HuCCT1 cells after 18 h incubation as indicated. (b) Flow cytometry analysis demonstrates that at concentrations of 10 *μ*M–20 *μ*M, both THC and CBN showed significantly increased apoptotic cells compared to the control (^*∗*^*P* < 0.001 versus the control cells; ^*a*^*P* < 0.001, 10 *μ*M THC versus 20 *μ*M THC; ^*b*^*P*=0.002, 10 *μ*M CBN versus 20 *μ*M CBN). (c) Cleaved PARP in HuCCT1 cells after treatment with 10 *μ*M–20 *μ*M of THC or CBN for 18 h was determined by western blotting. At a concentration of 20 *μ*M, THC and CBN induced cleaved PARP in HuCCT1 cells. PARP was used as a loading control.

Our study demonstrated that treatment with THC or CBN results in a reduction in cancer cell proliferation. We used an annexin V/PI assay to determine whether this reduction is associated with the induction of apoptosis. The results showed that the number of apoptotic HuCCT1 cells increased after the treatment of cancer cells with different concentrations of THC or CBN. The proportion of apoptotic cells increased from 24.01 ± 3.21% to 56.71 ± 4.63% with THC concentrations from 10 *μ*M to20 *μ*M (*P* < 0.001, 10 *μ*M THC versus 20 *μ*M THC), while the proportion increased from 25.43 ± 2.63% to 38.79 ± 4.28% with CBN concentrations of 10 *μ*M–20 *μ*M (*P*=0.002, 10 *μ*M CBN versus 20 *μ*M CBN) (Figures 4(a) and 4(b)).

The effect of THC and CBN on the apoptosis of CCA cells was confirmed by the detection of cleaved PARP. PARP is a nuclear DNA-binding protein that can detect DNA strand breaks and is involved in base excision repair. Once PARP is cleaved by caspase during apoptosis, its DNA repair function is impaired. The results showed that a band of 89 kDa, representing cleaved PARP, was clearly found in cells treated with THC or CBN at concentrations of 20 *μ*M (Figure 4(c)).

### 3.4. Effect of THC and CBN on Kinase Phosphorylated Proteins in CCA Cells

**Figure 5:**
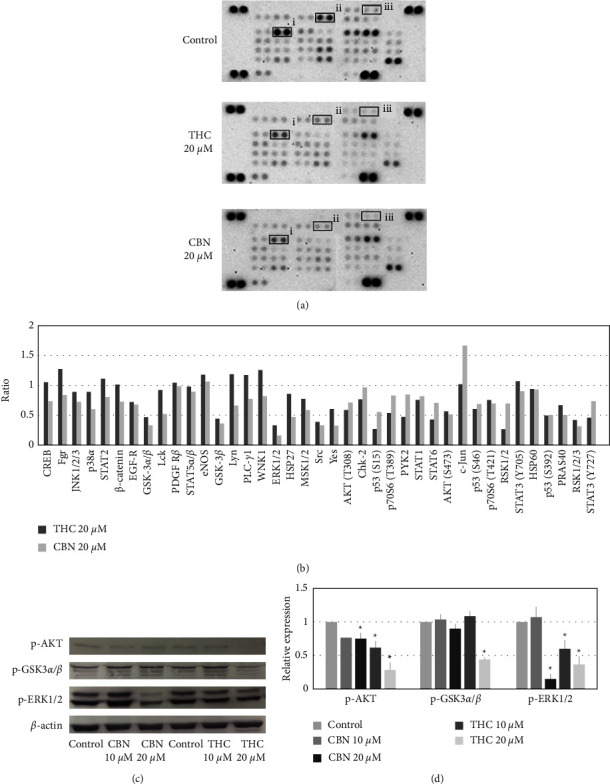
Effects of THC and CBN on CCA cell signaling. (a) The human phosphokinase array detected phosphorylated proteins in the control group, in HuCCT1 cells treated with 20 *μ*M of THC and in cells treated with 20 *μ*M of CBN after an 18 h incubation. Corresponding signals: (i) GSK-3*α*/*β* (phosphorylated at Ser21/9), (ii) ERK1/2 (phosphorylated at Thr202/Tyr204), and (iii) AKT (phosphorylated at Ser473). (b) Relative changes in phosphorylated kinase proteins between THC- or CBN-treated HuCCT1 cells and untreated HuCCT1 cells were demonstrated. (c) Selected phosphorylated kinase proteins in HuCCT1 cells after 10 *μ*M to 20 *μ*M THC or CBN treatment for 18 h were determined by western blotting. At a concentration of 20 *μ*M, THC and CBN inhibited the phosphorylation of AKT, GSK-3*α*/*β*, and ERK1/2. *β*-Actin was used as a loading control. (d) The relative levels of phosphorylation of AKT, GSK-3*α*/*β*, and ERK1/2 from western blot analyses were normalized to *β*-actin level (^*∗*^*P* < 0.01 versus control).

To study the signal transduction mediated by THC or CBN in CCA cells, the relative phosphorylation levels of 37 kinase phosphorylation sites and two related total proteins were simultaneously examined using the Proteome Profiler^TM^ Array and Human Phosphokinase Array Kit. Cells treated with THC or CBN demonstrated lower extents of phosphorylation of multiple signaling molecules than the control cells (Figures 5(a) and 5(b)). We selected phosphorylated proteins involved in cell proliferation and apoptosis in CCA [5] and confirmed these findings with western blot analyses.

The western blot results showed that the phosphorylation of AKT, GSK-3*α*/*β,* and ERK1/2 markedly decreased in a dose-dependent manner in HuCCT1 cells treated with THC or CBN. These findings suggest that treating HuCCT1 cells with THC or CBN inhibited the AKT and MAPK pathways (Figures 5(c) and 5(d)).

To determine whether these pathways are important for cell viability, we studied the proliferation of HuCCT1 cells after treatment with a specific inhibitor of AKT (Ly294002) or ERK1/2 (U0126). The results of the cell proliferation study demonstrated that inhibition of AKT or ERK1/2 with a specific inhibitor significantly decreased the rate of CCA cell proliferation (*P*=0.005 at 24 h and *P* < 0.001 at 48 h, 20 *μ*M Ly294002; *P*=0.018 at 24 h and *P* < 0.001 at 48 h, 20 *μ*M U0126) (Figure 3(a)).

### 3.5. Effect of THC and CBN on Tumor Xenograft Mice

**Figure 6:**
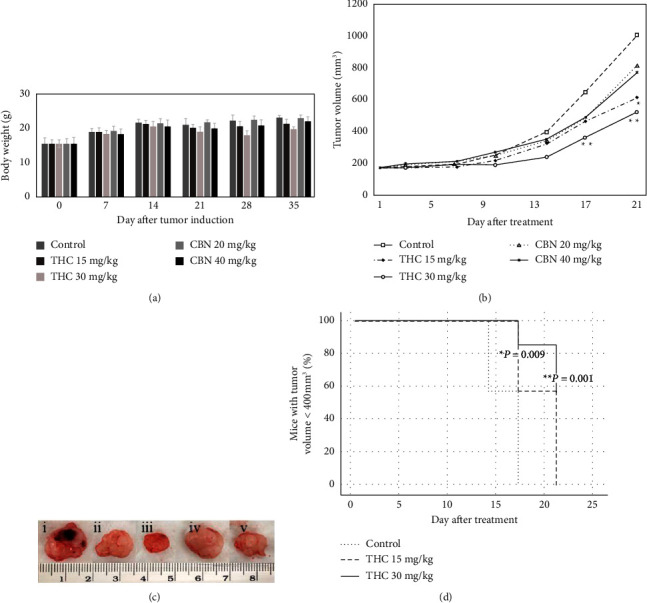
Effect of THC and CBN on tumor growth in HuCCT1 xenograft mice. (a) Development of the body weight of nude mice. (b) Tumor growth curve of control mice treated with 15 mg/kg THC, 30 mg/kg THC, 20 mg/kg CBN, and 40 mg/kg CBN. The growth of tumors in HuCCT1 xenograft mice treated with THC significantly decreased compared with control mice (^*∗*^*P*=0.006, ^*∗∗*^*P*=0.001 versus control mice). (c) Tumors of xenograft mice after 21 d of treatment with (i) control, (ii) 15 mg/kg THC, (iii) 30 mg/kg THC, (iv) 20 mg/kg CBN, and (v) 40 mg/kg CBN. (d) Survival curve indicating the percentage of mice with tumor volumes less than 400 mm^3^. THC concentrations of 15 mg/kg and 30 mg/kg significantly inhibited tumor growth in the HuCCT1 xenograft mice compared to the control mice (^*∗*^*P*=0.009 for 15 mg/kg THC and ^*∗∗*^*P*=0.001 for 30 mg/kg THC versus control mice).

After two weeks of nude mouse xenograft induction with HuCCT1 cells, the tumors were approximately 150 mm^3^ under the skin at the implantation site. THC, CBN, or control was injected around the tumor edge daily for three weeks. Nude mice in each group ate, drank, and excreted normally during the experiment. In addition, there was no significant difference between the average body weights of nude mice in the groups (Figure 6(a)).

From day 17 to day 21 of the interventional experiment, the tumor volume of the 30 mg/kg THC group was significantly smaller than that of the control group: on day 17, the average tumor volume in the 30 mg/kg THC group was 339.59 ± 65.26 mm^3^, while the average volume in the control group was 626.80 ± 113.46 mm^3^, *P*=0.001; on day 21, the average tumor volume in the 30 mg/kg THC group was 501.67 ± 109.06 mm^3^, while the average volume in the control group was 989.42 ± 236.09 mm^3^, *P*=0.001. On day 21, the tumor volume of the 15 mg/kg THC group was significantly smaller than that of the control group (the average tumor volume in the 15 mg/kg THC group was 594.56 ± 199.44 mm^3^, *P*=0.006). Meanwhile, nude mice treated with 20 mg/kg CBN and 40 mg/kg CBN exhibited decreased tumor volumes, but the difference was not statistically significant from the control group (*P* = 0.230 for 20 mg/kg CBN and *P*=0.144 for 40 mg/kg CBN on day 21) (Figures 6(b) and 6(c)). On day 17, all mice in the control group had tumor volumes greater than 400 mm^3^. Survival curves showed that mice treated with 15 mg/kg THC and 30 mg/kg THC had significantly slower tumor progression (assessed by the number of mice with tumor volumes  < 400 mm^3^) than the control group (*P*=0.009 for 15 mg/kg THC and *P*=0.001 for 30 mg/kg THC) (Figure 6(d)). Tumor progression in mice treated with 20 mg/kg CBN and 40 mg/kg CBN was not significantly different from tumor progression in control mice (*P*=1.000 for 20 mg/kg CBN and *P*=0.056 for 40 mg/kg CBN). This finding suggests that THC had an inhibitory effect on tumor growth in vivo.

### 3.6. Histopathological Study of CCA Xenograft Tumors

Tumor tissue sections revealed dense tubular and solid nests of adenocarcinoma that were well-circumscribed and encapsulated and that partially invaded the muscular layer, along with fine collagen fibers. The tumor cells were large and cuboidal to polyhedral, with light basophilic or amphophilic cytoplasm and large pleomorphic round to ovoid nuclei and one or two prominent nucleoli.

**Figure 7:**
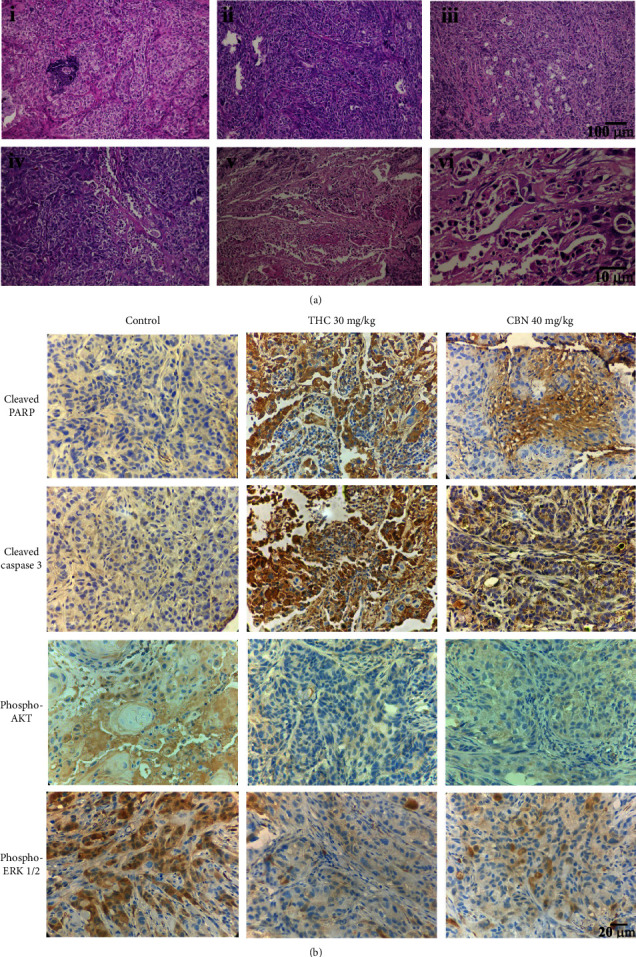
Representative H&E staining and immunohistochemistry of xenograft mouse specimens. (a) H&E staining of specimens from xenograft mice treated for 21 d with (i) control, (ii) 20 mg/kg CBN, (iii) 40 mg/kg CBN, (iv) 15 mg/kg THC, (v) 30 mg/kg THC (i–v, scale bars: 100 *μ*m), and (vi) 30 mg/kg THC (scale bar: 10 *μ*m). (b) Immunohistochemical staining of specimens from xenograft mice treated as indicated. The specimens of xenograft mice treated with 30 mg/kg THC or 40 mg/kg CBN markedly expressed cleaved PARP and cleaved caspase 3 but scarcely expressed phosphorylated AKT and ERK1/2 compared to the specimens of the control mice (scale bars: 20 *μ*m).

H&E staining showed multifocal intralesional cell necrosis. Mainly pyknotic and karyorrhectic nuclei were found in 10% of the specimens from the 15 mg/kg THC-treated group, 20% of the specimens from the 30 mg/kg THC-treated group, and 10% of the specimens from the 40 mg/kg CBN-treated group. No evidence of cell necrosis was found in tumor specimens of the control group or the 20 mg/kg CBN-treated group (Figure 7(a)). Immunohistochemical staining showed a significant increase of activated caspase-3 and downstream PARP cleavage in tumor specimens of the 30 mg/kg THC-treated group and the 40 mg/kg CBN-treated group, whereas these stained cells were rarely found in tumor specimens of the control group (Figure 7(b)).

Additionally, the phosphorylation of AKT and ERK1/2 was found to be significant in the tumor specimens derived from the control group. Meanwhile, the phosphorylation of AKT and ERK1/2 was dramatically decreased in tumor specimens of the THC- and CBN-treated groups (Figure 7(b)).

## 4. Discussion

To date, despite increased knowledge of CCA pathophysiology, only minor improvements in the treatment of this disease have been achieved. Therefore, identifying a novel treatment for CCA is important. THC and CBN share similar structural formulas. THC is the major psychoactive cannabinoid and shows various biological activities, including anticancer activity, in vitro and in vivo. CBN is found in trace amounts in the cannabis plant. CBN is mildly psychoactive, has anticonvulsant, sedative, anti-inflammatory, and antibiotic activities and promotes bone formation [18]. Therefore, the present study investigated the anticancer activities of THC and CBN in CCA in vitro and in vivo.

In this study, we demonstrate that both THC and CBN exhibited potent anticancer activity toward CCA in both cell culture and cancer xenograft mouse models. Both THC and CBN significantly inhibited the viability of CCA cells. Additionally, flow cytometric analysis using annexin V/PI showed that THC and CBN dose-dependently induced apoptosis in CCA cells. The effect of the cannabinoids on apoptosis in CCA cells was confirmed by western blot analysis. Cleavage of PARP 1 at Asp214 by caspase 3 is a useful marker of cell apoptosis. In this study, cleaved PARP was found in CCA cells treated with THC and CBN at a concentration of 20 *μ*M. This finding is consistent with previous studies in which THC-induced apoptosis in many kinds of cancer cells [21–24]. Interestingly, we demonstrated for the first time that CBN has antitumor activity.

Our experiments showed decreased phosphorylation of AKT in HuCCT1 cells treated with THC and CBN. Our previous work demonstrated that signal transduction of AKT is a crucial mechanism of CCA cell proliferation and invasion [14]. It has been proposed that AKT suppresses apoptosis by preserving Bcl-x activity and blocking mitochondrial cytochrome c release [25]. This finding is consistent with a previous study in which cannabinoids induced apoptotic cell death in glioma cells by inducing de novo synthesis of ceramide [26]. Ceramide accumulation leads to the activation of stress-related endoplasmic reticulum (ER) signaling and apoptosis pathways via AKT inhibition. We also found that the phosphorylation of GSK-3*α*/*β* at Ser21/9 decreased in HuCCT1 cells treated with THC. GSK-3*α*/*β* is a serine/threonine protein kinase that phosphorylates and inactivates glycogen synthase and regulates cyclin D1 proteolysis and subcellular localization. GSK-3*α*/*β* is a critical downstream signal of the AKT pathway [27, 28]. GSK-3*α*/*β* activity can be inhibited by AKT-mediated phosphorylation at Ser21 of GSK-3*α* and Ser9 of GSK-3*β* [27]. Therefore, we suggest that THC and CBN inhibit CCA cells by inhibiting AKT and its downstream signaling pathway.

Our previous study demonstrated that ERK1/2 signaling promotes CCA cell proliferation and that the inhibition of ERK1/2 contributes to cell apoptosis [29]. The phosphorylation of ERK1/2 was distinctly diminished after HuCCT1 cells were treated with THC and CBN both in vitro and in vivo. These findings contradict those of a previous study in which the activation of the CB1 receptor caused increased phosphorylation of ERK1/2. Another study suggested that CB1 receptor-induced ERK1/2 activation required AKT signal activation [30]. Our study and our previous work on RMCCA1 CCA cells found that the treatment of CCA with cannabinoids attenuated the phosphorylation of AKT and MAPK pathways [14]. Therefore, the effects of cannabinoids on ERK phosphorylation appear to be cell-type specific. To confirm that the AKT and MAPK pathways are important for CCA cell survival, specific inhibitors of AKT (LY294002) and ERK1/2 (U0126) were used in the cell proliferation assay. The results showed that LY294002 and U0126 significantly inhibited CCA cell proliferation. This finding is consistent with previous studies [31]. Therefore, we suggest that THC and CBN inhibit not only the AKT pathway but also the MAPK pathway to induce CCA cell apoptosis.

In addition, we discovered that cell migration was significantly reduced in CCA cells treated with THC or CBN. Previous research has shown that THC-inhibited cancer cell migration is dependent on the AKT and MEK signaling pathways [32]. As a result, we speculate that the suppression of cell migration by THC and CBN is due to the modification of the AKT and MEK signaling pathways. THC and CBN treated in CCA should be further explored for their antimigration and anti-invasion mechanisms.

To further extend the potential clinical applications, we tested the antitumor effect of THC and CBN on human CCA xenograft nude mice. The tumor volumes of mice treated with THC were significantly decreased compared to that of control mice. Although the in vitro study found that CBN distinctly inhibited CCA cells, the in vivo study demonstrated that the tumor volumes of mice treated with CBN were decreased but were not significantly different from that of the control group. However, an immunohistochemical study of tumor specimens showed that both THC and CBN induced cancer cell necrosis and apoptosis. Tumors from THC- and CBN-treated xenograft mice showed a reduction in phosphorylation of AKT and ERK1/2 in immunohistochemistry studies. This finding was associated with increases in cleaved PARP and cleaved caspase, both of which are markers of apoptosis.

This study demonstrates that while CBN showed anticancer activity in xenograft mice, it showed less anticancer activity than THC. CBN may have weaker affinity and specificity with CB1, CB2, and TRPV2 receptors than THC, resulting in distinct pharmacological effects [13, 18, 33]. Further study of cannabinoid receptors associated with the antitumor effects of THC and CBN in CCA should be performed.

These findings suggest that CBN has a weaker inhibitory effect on CCA xenograft mice than THC. Therefore, CBN could be synergistically applied with THC to inhibit CCA and prevent THC's psychotropic side effects. Further studies should address this possibility.

## 5. Conclusions

THC and CBN induced apoptosis in CCA by inhibiting the AKT and MAPK pathways, leading to a decrease in cell proliferation in vitro and tumor volume in vivo. In addition, in this animal model, THC appeared to be superior in potency to CBN. These findings provide a strong rationale for THC and CBN as therapeutic options for CCA.

## Data Availability

All the data used to support the result of this research can be obtained from the corresponding author upon reasonable request.
